# Multisite and multitimepoint proteomics reveal that patent foramen ovale closure improves migraine and epilepsy by reducing right‐to‐left shunt‐induced hypoxia

**DOI:** 10.1002/mco2.334

**Published:** 2023-08-12

**Authors:** Bosi Dong, Ying Lu, Siyu He, Baichuan Li, Yajiao Li, Qi Lai, Wanling Li, Shuming Ji, Yucheng Chen, Lunzhi Dai, Lei Chen

**Affiliations:** ^1^ Department of Neurology West China Hospital Sichuan University Chengdu Sichuan China; ^2^ State Key Laboratory of Biotherapy National Clinical Research Center for Geriatrics and Department of General Practice West China Hospital Sichuan University and Collaborative Innovation Center of Biotherapy Chengdu Sichuan China; ^3^ Department of Cardiology West China Hospital Sichuan University Chengdu Sichuan China; ^4^ Department of Clinical Research Management West China Hospital Sichuan University Chengdu Sichuan China

**Keywords:** epilepsy, hypoxia, migraine, patent foramen ovale

## Abstract

Patent foramen ovale (PFO) is a congenital defect in the partition between two atria, which may cause right‐to‐left shunt (RLS), leading to neurological chronic diseases with episodic manifestations (NCDEMs), such as migraine and epilepsy. However, whether PFO closure was effective in improving NCDEMs and the mechanism were unclear. Twenty‐eight patients with migraine or epilepsy who underwent PFO closure were recruited. Notably, approximately half of patients received 50% or more reduction in seizure or headache attacks. Meanwhile, the postoperative blood oxygen partial pressure and oxygen saturation were elevated after PFO closure. Multisite (peripheral, right, and left atrial) and multitimepoint (before and after surgery) plasma proteomics from patients showed that the levels of free hemoglobin and cell adhesion molecules (CAMs) were significantly increased after PFO closure, which may be related to the relief of the hypoxic state. Furtherly, the omics data from multiple brain regions of mice revealed that a large number of proteins were differentially expressed in the occipital region in response to PFO, including redox molecules and CAMs, suggesting PFO‐caused hypoxia may have great impacts on occipital region. Collectively, PFO may cause NCDEMs due to RLS‐induced hypoxia, and PFO closure could prevent RLS to improve migraine and epilepsy.

## Graphical abstract

Patent foramen ovale (PFO), a congenital heart disease causing right‐to‐left shunt, is considered as one etiological factor of neurological chronic diseases with episodic manifestations (NCDEMs). PFO closure can improve NCDEMs and relieve hypoxic state. Multisite and multitimepoint proteomics reveal the restoration of hypoxia‐associated proteome patterns after PFO closure. Multiomics analysis of multiple brain regions in PFO model shows the hypoxic state alters the expression of adhesion molecules in the occipital region, which may be responsible for PFO‐induced NCDEMs.

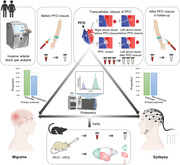

## 1 INTRODUCTION

Epilepsy and migraine are the two most common neurological chronic diseases with episodic manifestations (NCDEMs), which are collectively characterized by recurrent episodes of neurological dysfunction that return to baseline between episodes.^1^ Epilepsy and migraine, each occupying 20% of outpatient neurological visits, have similar symptom profiles, comorbidities, and treatment options, as well as common pathogenic mechanisms such as hypoxia, cortical spreading depression (CSD), and microemboli.^2^, ^3^ In addition, hypoxia can independently trigger ictaform activity, aura, photophobia, and phonophobia, which are common clinical symptoms of NCDEMs.^4^

Patent foramen ovale (PFO), resulted from failed primum and secundum septal fusion during the postnatal period, is one of the most common congenital cardiac malformations, with a prevalence of approximately 20% in the general population, as determined through autopsy studies.^5^, ^6^ Although most PFOs are clinically asymptomatic, recent studies have found that PFO, serving as a venous blood conduit causing right‐to‐left shunt (RLS), may be related to NCDEMs.^7^, ^8^ It has been reported that there are approximately 40% PFO positive individuals in the migraine and epilepsy group, which was much higher than normal population.^9^ The prevalence of PFO could increase the risk of migraine with aura in case–control studies.^10^ A causal relationship between PFO and migraine has been proved for the improvement of migraine frequency and severity after percutaneous PFO closure has been widely reported.^11^ Thus, PFO is known as “the back door to the brain”.^12^

There are several mechanisms being hypothesized and proposed. Micro‐emboli from deep venous thrombosis could directly enter arterial circulation by RLS leading to asymptomatic infarctions which might triggers ictaform activity.^13^ Some small molecules avoiding metabolism of lung by RLS, such as choline compounds, glutamine, reactive oxygen species (ROS), and serotonin, could also lead to NCDEMs.^14^, ^15^ Previous studies have also suggested that deoxygenated venous blood through PFO‐associated RLS may directly cause intermittent hypoxia and induce headache attacks.^16^ However, conclusive evidences for these hypotheses are lacking. Additionally, whether PFO is associated with epilepsy and the mechanism by which PFO affects epilepsy are unclear. To reveal the potential mechanism, plasma samples were obtained from the peripheral blood of patients with migraine (PWMs) and patients with epilepsy (PWEs) before and within a year after the operation, as well as from right and left atrial blood collected during surgery, for proteomics analysis. Multisite omics helps for finding common and difference of spatial expression,^17^ and multitimepoint omics could be helpful for searching unique changes detectable long after the resolution of perioperative state,^18^ providing PFO closure exerts profound protein‐expressed effects that can be traced in certain systems. Based on the multisite and multitimepoint proteomics and clinical data, as well as the omics data from PFO‐positive and PFO‐negative mice, we revealed that PFO‐induced RLS may evoke hypoxia. This study, for the first time, shows that PFO closure is beneficial for PWMs and PWEs through relieving hypoxia.

## 2 RESULTS

### 2.1 PFO closure improves migraine and epilepsy symptoms

To reveal the prognosis of NCDEM patients after PFO closure, we recruited 28 patients with NCDEMs who underwent PFO closure, including 16 PWEs and 12 PWMs who were followed up for 19.43 ± 5.21 months. The median age of these 28 subjects was 25.43 years, and 13 (46.4%) were male. The age at the onset of epilepsy was 13.56 ± 6.51 years, and the disease duration before PFO closure was 7.69 ± 4.69 years; in addition, the age at onset of migraine was 29.42 ± 10.50 years, and the disease duration prior to PFO closure was 8.75 ± 7.70 years (Table 1). On the first day postoperation, the cardiac occluders in all patients were confirmed to be in a correct position by chest radiographs and TTE. There were no adverse effects resulting in prolonged hospital stays. No adverse reactions were reported during follow‐up.

**TABLE 1:** Clinical information for the participants.

	PWMs (*n* = 12)	PWEs (*n* = 16)
Gender (male,%)	3 (25.0)	10 (62.5)
Onset age (y, mean ± SD)	29.42 ± 10.50	13.56 ± 6.51
RLS grade (III, %)	9 (75.0)	12 (75.0)
Age at PFO closure (y, mean ± SD)	38.00 ± 11.08	21.25 ± 5.39
With aura (n, %)	3 (25.0)	7 (43.8)
Intractable migraine or epilepsy (n, %)	2 (16.7)	11 (68.8)
Following up (month, mean ± SD)	17.00 ± 3.63	21.25 ± 5.46
More than 50% reduction in frequency of attacks (*n*, %)	7 (58.3)	8 (50.0)

PWEs, patients with epilepsy; PWMs, patients with migraine; PFO, patent foramen ovale; RLS, right‐to‐left shunt.

**FIGURE 1:**
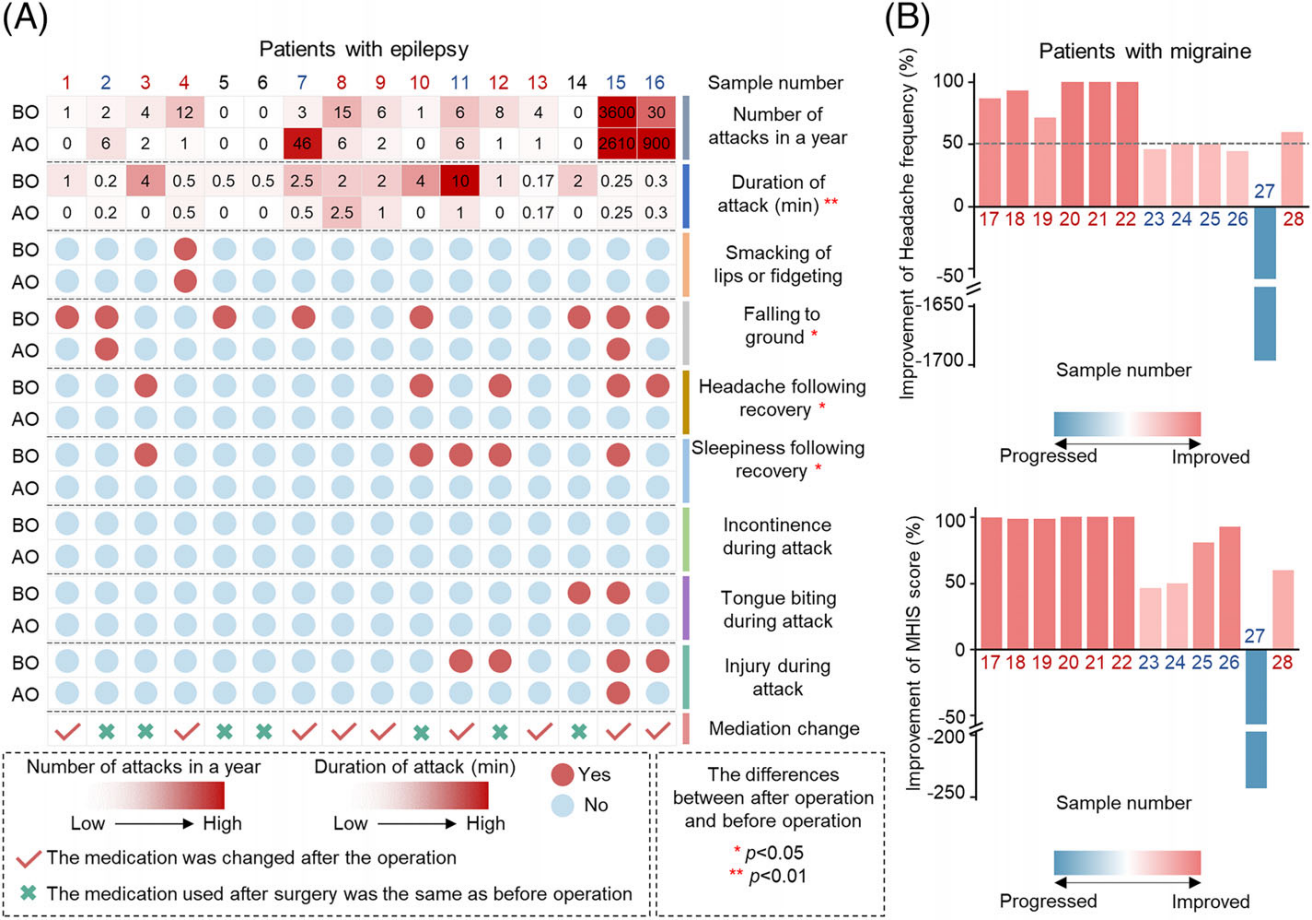
Improvements in epilepsy and migraine symptoms after PFO closure. (A) Frequency, duration, severity of seizures, and ASM usage before and after PFO closure in PWEs (Fisher exact test for categorical variables and Mann–Whitney *U* test for continuous data). *p < 0.05, **p < 0.01. The deeper the red, the more seizure attacks in a year and longer duration of attacks. Red color dot represents existence of the ictal symptom, and blue color dot represents nonexistence of the ictal symptom. (B) Improvement of headache attacks in PWMs after PFO closure. PWMs, patients with migraine. A more than 50% reduction in frequency of seizures or headache attacks compared with that before PFO closure was considered an improvement. Numbers from the first to the 16th are for epileptic patients, and numbers from the 17th to the 28th are for migraine patients. Numbers in red font indicate patients who have improved, numbers in dark blue represent patients who did not improve, and numbers in black font indicates that the improvement cannot be confirmed. A more than 50% reduction in seizures or headache attacks compared with that before PFO closure was considered a significant improvement. PWEs, patients with epilepsy; PWMs, patients with migraine; PFO, patent foramen ovale; BO, before operation; AO, after operation; ASM, antiseizure medication; MHIS, migraine headache index score.

The frequency of seizures or headaches before and after PFO closure was measured in PWEs and PWMs, as shown in Figure 1. A more than 50% reduction in seizures or headache attacks compared with that before PFO closure was considered a significant improvement.^19^, ^20^ Among PWEs, nine (56.3%) exhibited reduced seizure frequency after PFO closure (Figure 1A). Of these nine patients, eight (50.0%) experienced a decrease in seizure frequency of greater than 50%, and of these eight patients, three with refractory epilepsy showed an improvement in symptoms without antiseizure medication (ASM) adjustment. Moreover, there were significant differences in the degree to which epilepsy duration. After PFO closure, seven PWMs (58.3%) experienced relief, but the remaining five showed no significant improvement in migraine attacks (Figure 1B). Collectively, these data suggest that PFO closure could improve migraine and epilepsy symptoms.

### 2.2 Plasma free hemoglobin levels increase after PFO closure

To explore the relationship between PFO and migraine and epilepsy, proteomics study was carried out using plasma samples collected at multiple time points and from multiple sampling locations in those 28 patients before and after PFO closure, including 24 preoperative left atrial plasma, 25 preoperative right atrial plasma, 19 preoperative peripheral plasma, 24 postoperative right atrial plasma, and 28 postoperative peripheral plasma. Peripheral venous plasma was collected an average of 101.84 days before the operation and 141.43 days after the operation.

**FIGURE 2:**
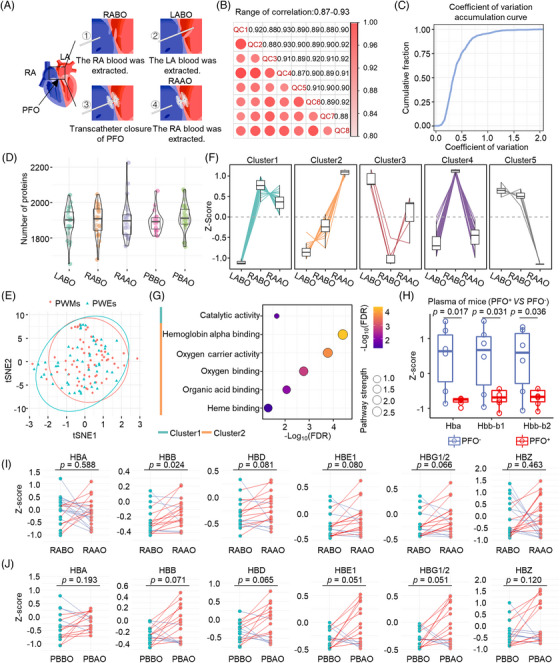
Free hemoglobin levels increase after PFO closure. (A) Schematic diagram of the atrial blood collection process during the operation. (B) Pearson correlation analysis of quality controls. (C) Coefficient of variation accumulation curve for quality control. (D) Number of proteins identified in each sample of five blood positions. (E) A tSNE analysis for the visualization of proteomics data of PWEs and PWMs. (F) Cluster analysis for differential expression of proteins between atrial and peripheral plasma. (G) Enrichment analysis of proteins in each cluster. (H) The levels of free hemoglobin variants in PFO‐negative (*n* = 6) and PFO‐positive (*n* = 5) mice were shown by boxplot. (I and J). The plot presents the levels of free hemoglobin variants in the right atrium blood and peripheral blood. Each line connects the same patients before and after PFO closure. QC, quality controls; LABO, blood of the left atrium before the operation; RABO, blood of the right atrium before the operation; RAAO, blood of the right atrium after the operation; PBBO, peripheral blood before operation; PBAO, peripheral blood after operation; HBA, hemoglobin subunit alpha; HBB, hemoglobin subunit beta; HBD, hemoglobin subunit delta; HBE, hemoglobin subunit epsilon; HBG, hemoglobin subunit gamma; HBZ, hemoglobin subunit zeta.

Because PFO causes abnormal hemodynamics in RLS, resulting in venous blood directly entering the arterial circulatory system, in addition to peripheral venous plasma, we also collected the plasma of the right atrium and left atrium before and after occlusion for proteomics analysis following the approach shown in Figure 2A. During mass spectrometry (MS) detection, continuous quality controls (QCs) were analyzed every 20 MS injections to monitor the stability of the machine. We found good correlation between the QC samples (0.87–0.93; Figure 2B), and more than 75% proteins had coefficient of variation less than 50% (Figure 2C). The number of proteins identified in each sample of five blood positions was shown in Figure 2D. A t‐stochastic neighbor embedding (tSNE) analysis for the visualization of proteomics data of PWEs and PWMs showed there was no clear distinction for the PWEs and PWMs (Figure 2E) and subgroup analyses of PWEs and PWMs showed in Figure S1A and S1B, respectively. To capture the changes caused by PFO closure, only patients with a full set of preoperative and postoperative samples were included in proteomics analysis. We identified 66 differentially expressed proteins in the comparison of plasma samples from the left atrium before PFO closure and from the right atrium before and after PFO closure. These 66 proteins were clustered into five clusters (Figure 2F) and enrichment analysis of the proteins in each cluster was carried out to measure the dissimilarity (Figure 2G). The details of Gene Ontology (GO) enrichment results are shown in Table S1. Of note, oxygen transport was most significantly enriched in cluster 2 (Figure 2G). Compared with samples from the right atrium and veins, samples from the left atrium had increased blood oxygen levels but decreased free hemoglobin levels (Figure 2F). However, the levels of most free hemoglobin variants in plasma from the right atrium of PWEs and PWMs increased after PFO closure (Figure 2I). In addition, the levels of free hemoglobin variants were also increased in the peripheral blood after PFO closure (Figure 2J). We further compared the proteome difference between patients with different response after PFO closure. We found that hemoglobin in the blood of the right atrium increased more significantly after PFO closure and oxygen transport also enriched in the improvers (Figures S2A and S2B). Collectively, free hemoglobin levels increased after PFO closure.

To demonstrate whether the levels of free hemoglobin variants are related to PFO, we collected the plasma of PFO‐positive and PFO‐negative mice. As shown in Figure S1, the positive mouse model presented a clear PFO between right atrium and left atrium. We analyzed the levels of free hemoglobin in plasma from the peripheral blood of PFO‐positive and ‐negative mice and found higher in PFO‐negative mice than in PFO‐positive mice (Figure 2H). Hypoxia can reduce the utilization of nitric oxide (NO), which can modulate synaptic transmission,^21^ and an increase in NO levels has been observed during epileptic activity^22^ and contributes to migraine.^23^ Interestingly, free hemoglobin is 600 times more capable of removing NO than hemoglobin in erythrocytes.^24^ Oxygen transport pathway and oxygen transporter activity are also related to neurological deficiencies.^25^ Therefore, increased levels of free hemoglobin variants after PFO closure may be beneficial for migraine and epilepsy patients.

### 2.3 Elevated enrichment levels of plasma cell adhension molecules (CAMs) after PFO closure

**FIGURE 3:**
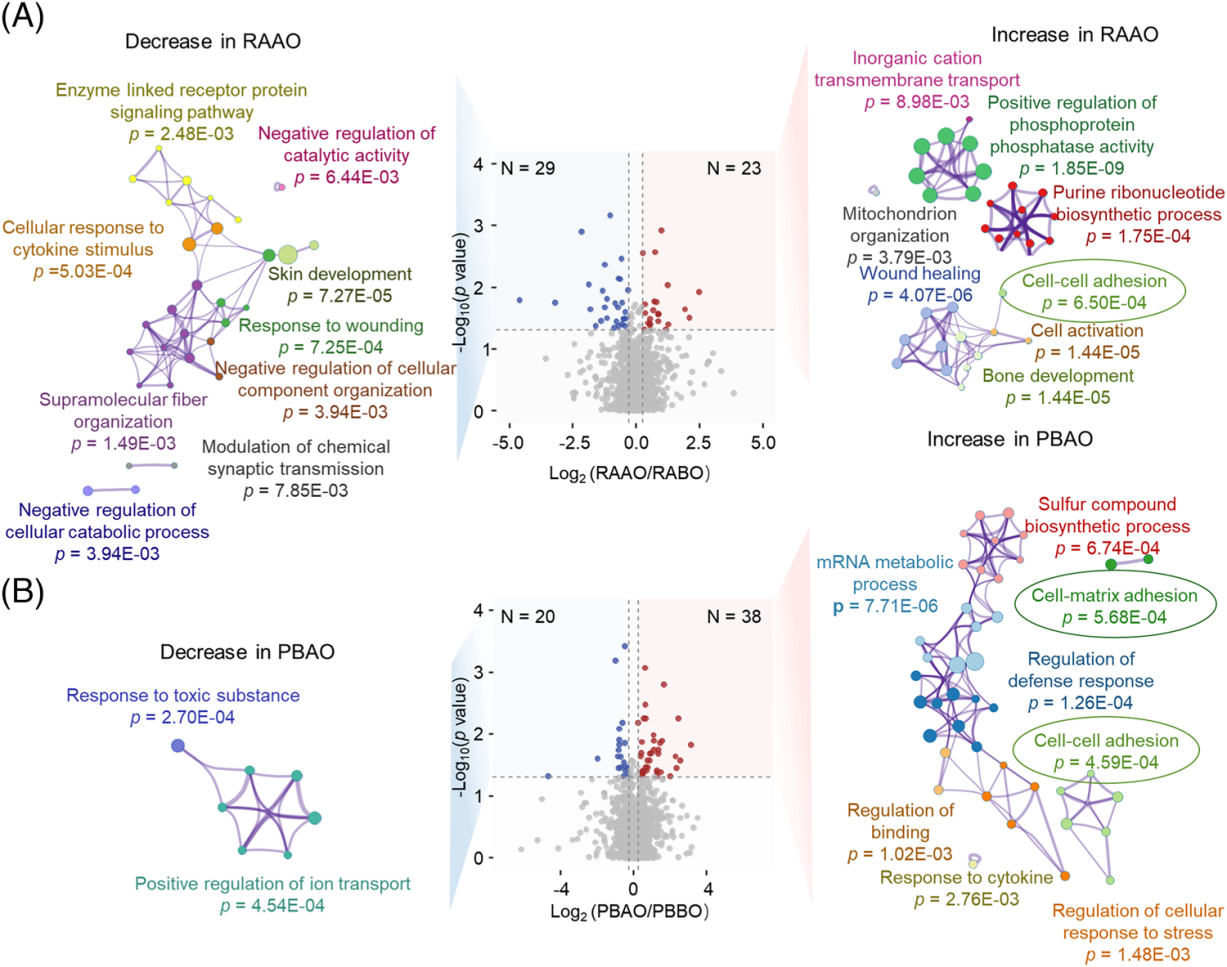
Changes in plasma cell adhesion molecules after PFO closure in patients. (A) Enrichment analysis of differentially expressed proteins in the right atrium preoperation and postoperation. (B) Enrichment analysis of differentially expressed proteins in peripheral blood preoperation and postoperation. The red and blue dots represent that the proteins are upregulated and downregulated after operation, respectively. The pathways related to cell adhesion are circled in the figure.

We further analyzed the proteomics differences in the right atrial and venous blood of patients before and after PFO closure. A total of 65 right atrial differential proteins and 68 venous differential proteins were detected after PFO closure, seven of these differentially expressed proteins were identified in both comparisons. To verify function of those differentially expressed proteins, pathway enrichment of the differentially expressed proteins in the right atrium after PFO closure was performed (Table S2). This analysis identified the significant downregulation of the enzyme‐linked receptor protein signaling pathway, cellular response to cytokine stimulus, and modulation of chemical synaptic transmission, as well as the significant upregulation of mitochondrion organization, wound healing, purine ribonucleotide biosynthetic process and cell‒cell adhesion (Figure 3A). In peripheral blood after PFO closure, the response to toxic substances and positive regulation of ion transport were significantly downregulated, while cell matrix adhesion and cell‒cell adhesion were significantly upregulated (Figure 3B). Notably, increased levels of CAMs were observed in both atrial and venous blood after the operation. Specifically, CAMs upregulated after PFO closure included Contactin 1 (CNTN1), Hemoglobin subunit Beta (HBB), Protein Tyrosine Phosphatase Receptor (PTPR) type C and SPARCL1 in right atrial blood, and Activated Leukocyte Cell Adhesion Molecule (ALCAM), Cadherin 1 (CDH1), PTPR type F, Collagen Type XIV Alpha 1 Chain (COL14A1), Talin 2 (TLN2), Adenosine Deaminase RNA Specific B1 (ADARB1), Zyxin (ZYX), Fibulin 5 (FBLN5), and Lymphatic Vessel Endothelial Hyaluronan Receptor 1 (LYVE1) in peripheral blood. Additionally, proteins related to extracellular single‐regulated kinase (ERK) 1 and ERK2 cascade pathway, which can regulate cell adhesion and participate in neuroprotection,^26^, ^27^ increased significantly after PFO in improvers (Figure S2C). As changes in plasma CAMs are associated with the hypoxic state,^28^, ^29^ PFO closure may alter the levels of CAMs in plasma by affecting hypoxia.

### 2.4 Blood gas analysis indicates an improved hypoxic state after PFO closure

**FIGURE 4:**
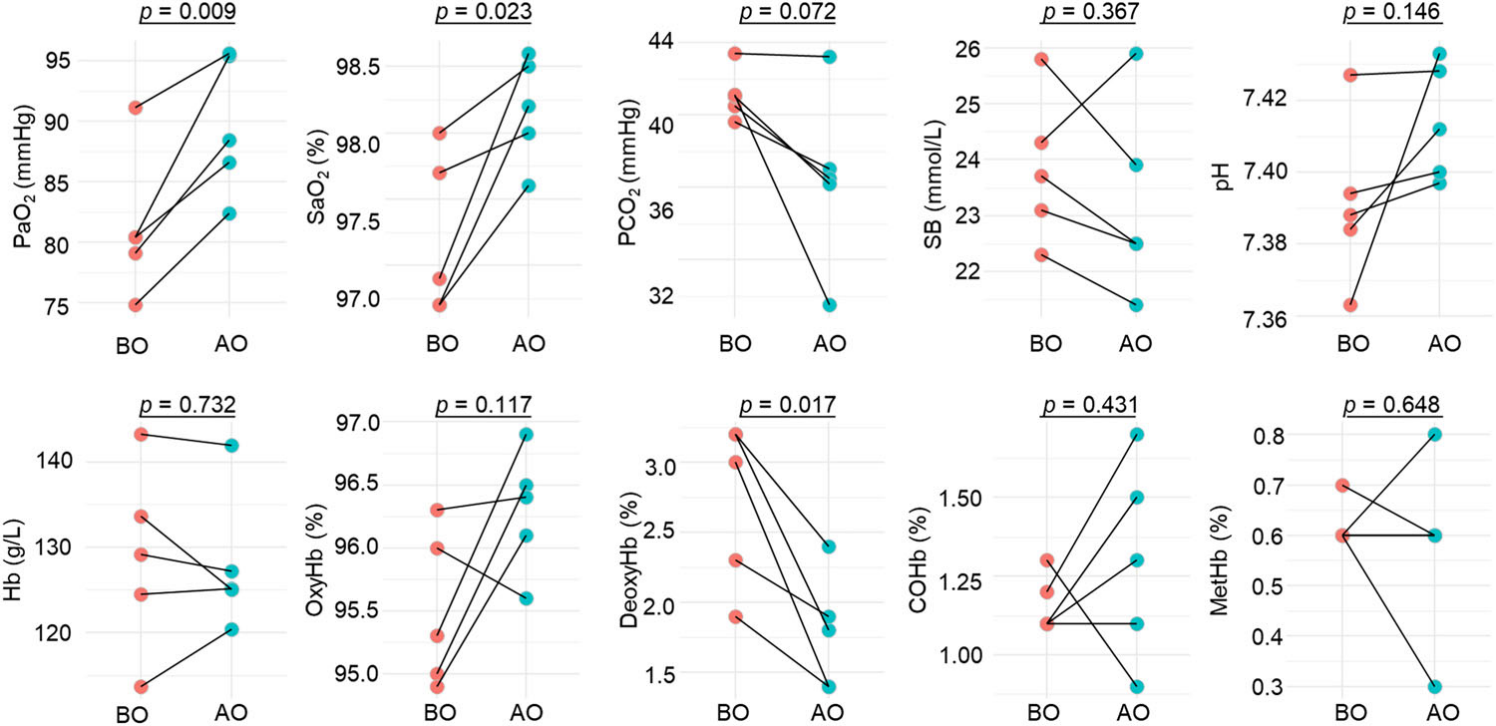
Arterial blood gas analysis before and after PFO closure. The differences of these blood gas indexes before and after operation were analyzed by the Student's *t*‐test. PaO_2_, partial pressure of arterial oxygen; SaO_2_, arterial oxygen saturation; PCO_2_, partial pressure of arterial carbon dioxide; SB, secretion of bicarbonate ions; Hb, hemoglobin; OxyHb, oxyhemoglobin; DeoxyHb, deoxyhemoglobin; COHb, carbohemoglobin; MetHb, methemoglobin; BO, before operation; AO, after operation.

To further demonstrate the relationship between PFO and hypoxia in NCDEMs, we performed arterial blood gas analysis before and after PFO closure in five patients who were willing to have arterial blood gas tests before PFO closure and re‐examine in one year after operation. The clinical characteristics of patients underwent preoperative and postoperative blood gas analysis were shown in Table S3. We found that the preoperative blood oxygenation in patients with PFO was low but within the normal range on admission (Figure 4). We compared the preoperative and postoperative blood gas results and found a significant increase in postoperative blood oxygen partial pressure (81.16 ± 5.38 versus 89.68 ± 5.14, *p* = 0.009) and oxygen saturation (97.20 ± 0.54 versus 98.18 ± 0.36, *p* = 0.023). The percentage of deoxyhemogolobin decreased after PFO closure (2.72 ± 0.53 versus 1.78 ± 0.37, *p* = 0.017), while the percentage of oxyhemoglobin, carbohemoglobin and methemoglobin, and concentration of total hemoglobin had no significant changes. Additionally, carbon dioxide partial pressure and bicarbonate concentration were also decreased, but not significantly (Figure 4). Partial oxygen pressure and oxygen saturation are important and sensitive parameters of hypoxia,^30^ and neurons are the most sensitive to hypoxia among the cells.^31^ This analysis of arterial blood gas before and after surgery showed a certain degree of hypoxia in individuals with PFO that could be relieved after PFO closure, which is beneficial for patients with NCDEMs.

### 2.5 RLS has a greater impact on protein expression in the occipital region

**FIGURE 5:**
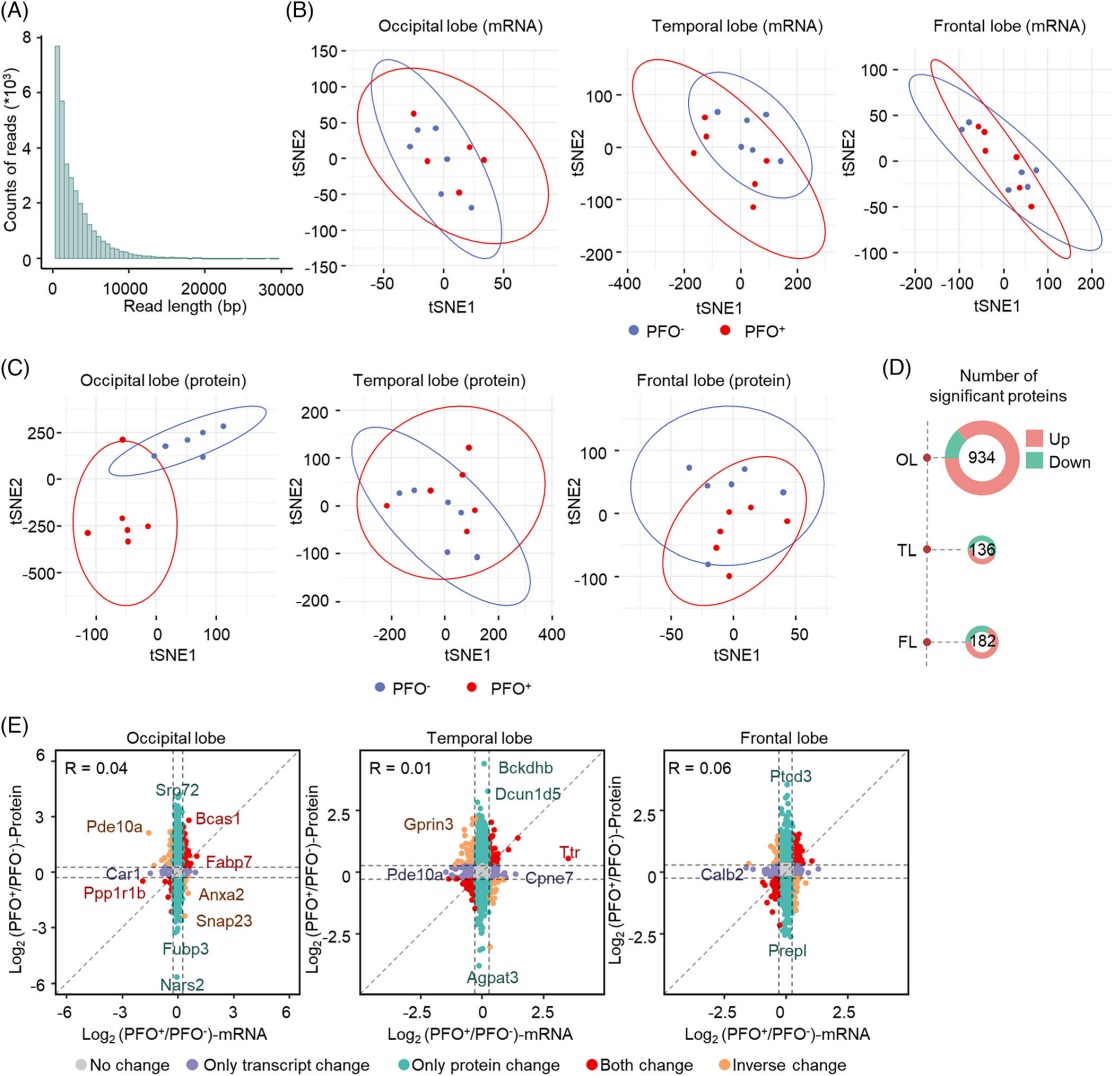
Proteome and transcriptome analysis of the frontal, temporal and occipital regions of PFO‐positive and PFO‐negative mice. (A) Quality control of the transcriptome. (B) Dimension reduction analysis of the transcriptome in mouse brain tissues. (C) Dimension reduction analysis of the proteome in mouse brain tissues. (D) Number of significantly differentially expressed proteins in the frontal, temporal and occipital regions. The significantly differentially expressed proteins were analyzed by Student's *t*‐test. (E) Correlation analysis of the proteome and transcriptome. tSNE, t‐stochastic neighbor embedding; FL, frontal lobe; TL, temporal lobe; OL, occipital lobe.

Since the left atrium receives systemic venous blood through RLS, and may indirectly reflect changes in the brain, we next explored whether PFO affects the expression of the brain. To answer this question, we investigated the molecular changes in three brain regions, including the occipital, temporal, and frontal regions, in 129T2/SvEms mice, which exhibit a high number of PFO phenotypes.^32^ QC of the transcriptome is shown in Figure 5A. Based on t‐SNE analysis of the proteome and transcriptome, we found that the transcriptome did not significantly differ in these three brain regions of PFO‐negative and PFO‐positive mice, but there were significant differences among these mice in the proteome of the occipital region (Figures 5B and C). Specifically, 934, 136, and 182 differentially expressed proteins between PFO‐negative and PFO‐positive mice were identified in the occipital, temporal and frontal regions, respectively (Figure 5D), suggesting that PFO more strongly affects the occipital region than the other two brain regions. As the correlation between the proteome and the transcriptome in the three brain regions was low (Figure 5E), the differences in the proteome may have resulted from changes at the translational and posttranslational level. Regional ischemia and excessive oxidative stress in the posterior head are often observed in stroke or migraine patients with PFO, which was shown in animal models to be associated with the development of CSD.^33^ CSD is a phenomenon that extends from the initial site of excessive neuronal excitability to the surrounding tissues and causes persistent suppression of cortical electrical activity, which often initially occurs in the posterior region of the head.^34^ This is one of the common pathogenic mechanisms underlying epilepsy and migraine.^35^ Our findings suggest that the posterior region of the brain is the main area responsible for PFO‐induced neurologic ictal disorders.

### 2.6 Occipital levels of redox‐associated proteins increase in PFO‐positive mice

**FIGURE 6:**
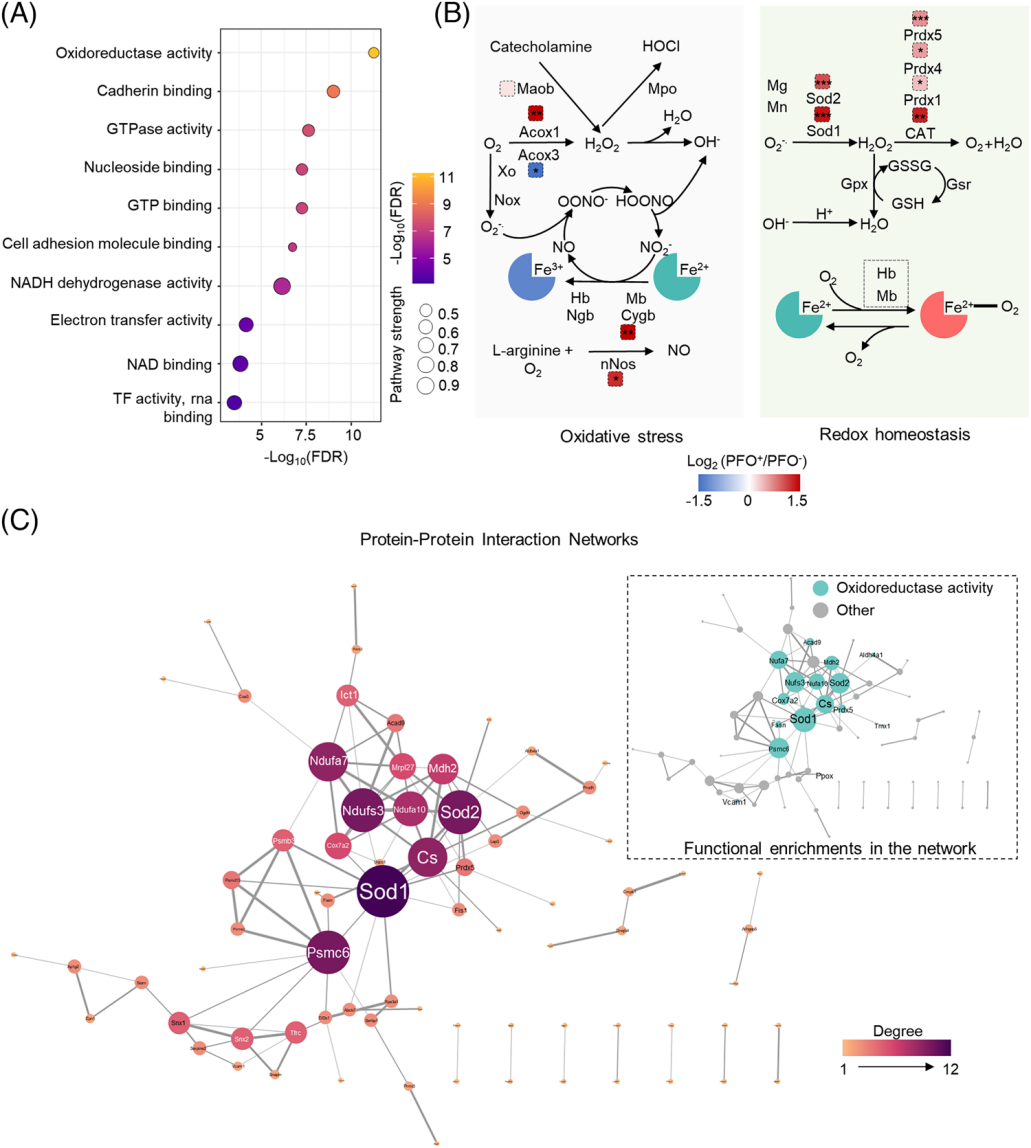
Differences in redox state of the occipital region between the PFO‐positive and PFO‐negative groups. (A) Enrichment analysis of differentially expressed proteins in the occipital region. (B) The significantly differentially expressed proteins related to redox in the occipital region between the PFO‐negative and PFO‐positive groups are shown in the pathway graph. The red and blue boxes indicate increases and decreases of proteins in PFO‐positive mice relative to PFO‐negative mice. The differences of proteins were analyzed by Student's *t*‐test. Oxidative stress (OS) refers to a state where oxidation and antioxidant effects are out of balance, which tends to be oxidative. OS produces a large number of oxidized intermediates, including reactive oxygen species (ROS) and reactive nitrogen species (RNSs). In order to neutralize excess ROS, a redox system based on enzymes and antioxidant molecules is activated. (C) The PPI network of the more significantly upregulated genes in PFO+ mice (*p* < 0.001, PFO+/PFO− > 1.2, *n* = 90) generated by the STRING database and visualized by Cytoscape software. The size and color of the node represent the number of other genes that are adjacent to it of the protein. The width of the edge represents the combined score between any pair of proteins. *p < 0.05, **p < 0.01, ***p < 0.001.

Next, we explored PFO‐induced proteomic changes in the posterior regions of the mouse brain to further explore expressed changes of brain brought by RLS. We found that 809 proteins increased and 125 proteins decreased in the occipital region of PFO‐positive mice than in that of PFO‐negative mice. In addition, gene ontology molecular function enrichment analysis identified significant increases in molecular functions associated with oxidoreductase activity, cadherin binding, GTPase activity, nucleoside binding, GTP binding, and CAM binding, with the most significant increase in oxidoreductase activity (Figure 6A), and detailed comments on the GO enrichment results are shown in Table S4. The tissue redox status related to GTPase function plays an important role in the development of NCDEMs.^36^ Notably, the occipital region of the brain in PFO‐positive mice showed increased levels of Acyl‐CoA Oxidase 1 (ACOX1), Nitric Oxide Synthase 1 (NOS1), Cytoglobin, Superoxide Dismutase (SOD) 1, SOD2, Catalase, Peroxiredoxin (PRDX) 1, PRDX4, and PRDX5, which are associated with oxidation and reduction (Figure 6B). Protein–protein interaction (PPI) analysis of significantly upregulated proteins showed a significant PPI enrichment in oxidoreductases (Figure 6C). Previous studies have shown that PFO can lead to intermittent hypoxia in the brain,^37^, ^38^ resulting in oxidative stress.^39^ These results suggest that intermittent hypoxia caused by PFO can induce oxidative stress responses in the occipital region and that increased levels of redox‐associated proteins may protect the brain.

### 2.7 Elevated enrichment levels of occipital CAMs in PFO‐positive mice

**FIGURE 7:**
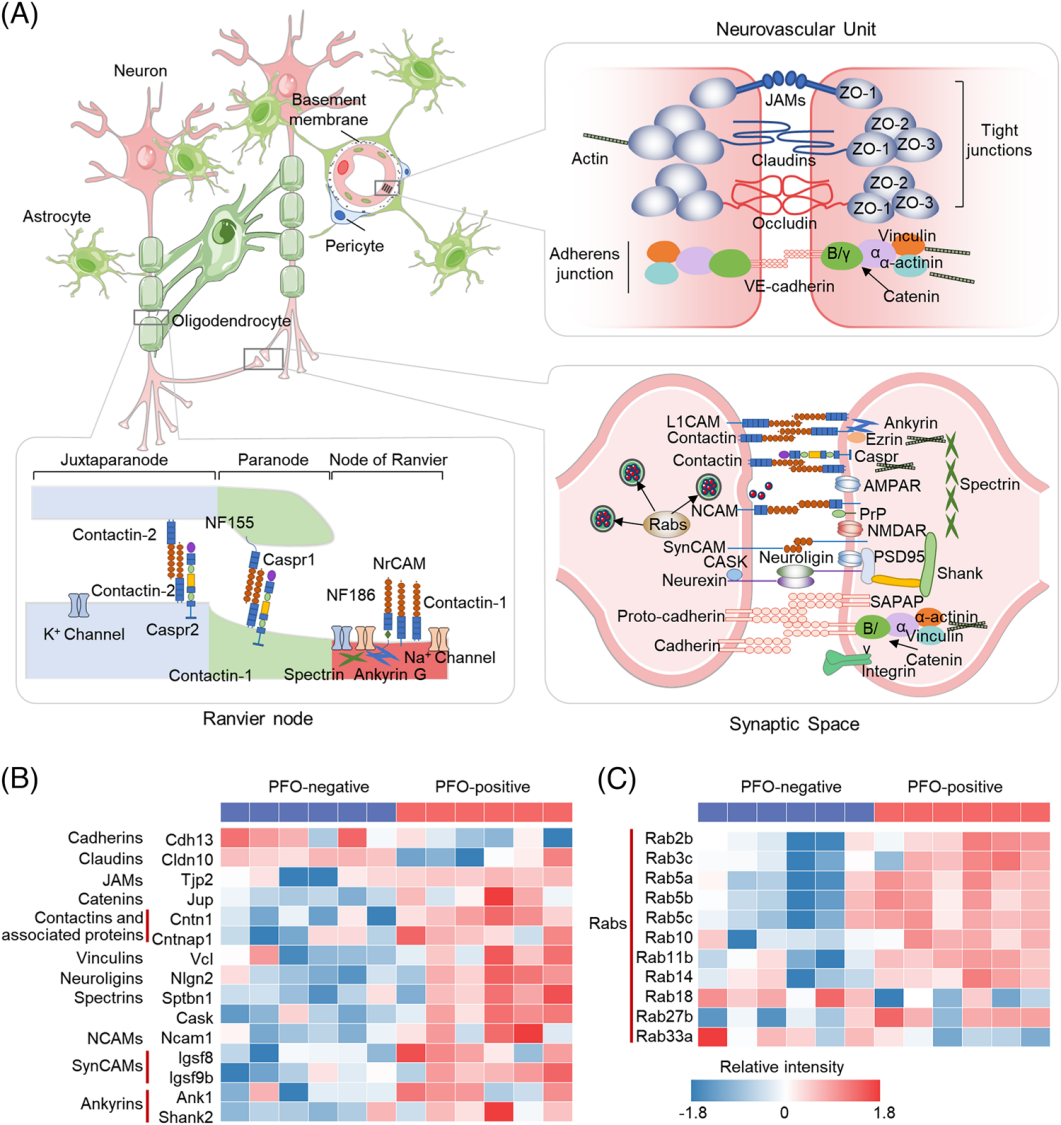
The levels of most adhesion and junction proteins are elevated in the occipital region of PFO‐positive mice. (A) Schematic of important adhesion molecules in the brain. In the brain nervous system, cell‐cell adhesion and junction molecules mainly play an important role in the blood–brain barrier, synapses, and Ranvier nodes. The figure shows the main adhesion and junction modes of each position. (B) Heatmap of the levels of significant adhesion and junction proteins in the occipital region of PFO‐negative and PFO‐positive mice. (C) Heatmap of the levels of significant Rab proteins in the occipital region of PFO‐negative and PFO‐positive mice. The differences of proteins were analyzed by Student's *t*‐test. JAMs, junctional adhesion molecules; NCAM, neural cell adhesion molecule; SynCAM, synaptic cell adhesion molecule; NMDAR, N‐methyl‐d‐aspartate receptor; CASK, calcium‐dependent serine protein kinase.

In addition to the levels of redox‐associated proteins, proteins related to cadherin binding and CAM binding significantly changed in response to PFO in the occipital region in mice (Figure 6A). Figure 7A shows schematic of important adhesion molecules in the brain. A large number of adhesion, cell–cell junction, and trafficking proteins in the nodes of Ranvier, neurovascular units, and synaptic spaces were significantly increased in the occipital region of PFO‐positive mice. For example, neural cell adhesion molecules (NCAMs) such as Ncam1 and synaptic cell adhesion molecules (SynCAMs) such as Igsf8 and Igsf9b, which are important regulators of synaptic plasticity before hypoxic‐ischemic neuronal cell death,^40^ were increased in the occipital region of PFO‐positive mice (Figure 7B). Neuroligin 2 (Nlgn2), a neuroligin located at inhibitory synapses that promotes inhibitory neuronal transmission,^41^ thereby causing glutamate‐related central sensitization through the GABABR2/SynCAM1 pathway,^42^ was also increased in PFO‐positive mice (Figure 7B). A previous study showed that Nlgn2 expression is significantly increased under low oxygen conditions in granulosa cells.^43^ In addition, the levels of members of the Rab family, which are key regulators of vesicular trafficking, were elevated in the occipital region of PFO‐positive mice (Figure 7C). These proteins included Rab3, which is related to Ca^2+^‐triggered neurotransmitter release; Rab5, which is associated with the promotion of neuronal polarization; and Rab27, which is involved in the maturation of circulating vesicles and their transport to the presynaptic plasma membrane.^44^, ^45^ Contactins are a class of adhesion molecules containing the neuro‐immunoglobulin domain.^46^ High expression of Cntn1 is associated with the aggregation of voltage‐gated sodium channels (Navs), which are essential for transmission along myelinated fibers and action potential regeneration, at the nodes of Ranvier.^47^ HIF‐1α can be induced by hypoxia and this transcription factor can regulate the expression of Cntn1,^48^ suggesting that PFO‐induced hypoxia may affect Cntn1 expression. Furthermore, the levels of some adhesion molecules related to the neurovascular unit were decreased in PFO‐positive mice (Figure 7B) including Cdh13 and Cdh10, which are important for maintaining the integrity of the blood‒brain barrier (BBB). The decrease in the levels of adhesion molecules in the neurovascular unit may cause destruction of the BBB,^49^ which has been demonstrated in migraine and epilepsy mouse models.^50^, ^51^ Collectively, PFO‐induced changes in occipital CAMs may contribute to migraine and epilepsy pathophysiology.

## 3 DISCUSSION

In summary, we report for the first time that PFO with RLS may cause hypoxia that induces migraine and epilepsy and that PFO closure can improve epilepsy symptoms, similar to the efficacy of PFO closure on migraine and cryptogenic stroke. Mechanistically, PFO‐induced hypoxia increases oxidative stress and the levels of CAMs in some brain regions, especially the occipital region, which is associated with CSD‐related NCDEMs. PFO closure prevents RLS and relieves hypoxia, which may restore proteome homeostasis in the brain and improve epilepsy and migraine.

PFO is a normal variant of the atrial septum accompanied by RLS, which enables unoxygenated blood to bypass pulmonary circulation and directly enter the arterial system^52^; this can lead to hypoxia in the brain and cause neurological deficits.^53^ Insufficient oxygen supply during CSD can cause clinical manifestations associated with the occipital cortex, such as long‐term contrast discrimination, prolonged visual evoked potential, reduced visual field sensitivity, visual aura, and other manifestations of migraine.^54^, ^55^ PFO closure can increase oxygen supply, which is important to shorten the duration of CSD, improve the local redox state of the brain,^56^ and prevent epilepsy and migraine. In accordance with previous data, our clinical data provide preliminary evidence for a significant increase in blood oxygen partial pressure and improvements in epilepsy and migraine after PFO closure. Therefore, we believe that PFO may cause hypoxia state of occipital region in occurrence of seizures or headache.

Hypoxemia mediates oxidative stress in the brain, which is associated with the occurrence of migraine and epilepsy.^57^ Several lines of evidence suggest that the immediate precipitant of seizure or headache attack is an acute rise in brain oxidative stress caused by nearly all provoked factors.^58^ For example, stimuli including noise, sleep deprivation, and psychosocial stress can generate ROS, which threaten neuronal viability and elicit migraine or epilepsy.^59^ ROS also play an important role in the development of refractory epilepsy, which likely occurs through the upregulation of P‐glycoprotein.^60^ Furthermore, previous animal research has revealed that seizure or headache disorders appear when the ventilatory response to hypercapnia or hypoxia is weak since pH and electrolyte imbalances are induced by oxidative stress.^61^ Hypoxia elevates redox signaling and oxidative stress that affect the spreading depolarizations in the occipital cortex that underlie the neurobiology of migraine with aura,^62^, ^63^ which is consistent with our finding of PFO‐induced changes in redox‐associated proteins in the occipital region.

Changes in oxygen supply also alter the expression of adhesion molecules.^29^ Adhesion molecules in the brain are crucial to the correct assembly and molecular composition of presynaptic and postsynaptic specializations and work together in nerves to maintain a balance between excitability and inhibition.^64^ In this work, we identified significant changes in adhesion molecules in response to PFO, which may contribute to migraine and epilepsy. Previous studies have found that tight junction proteins at the BBB are redistributed under hypoxia–reoxygenation conditions,^65^ and the upregulation of tight junction protein 2, vinculin, and N‐cadherin enhances antipermeability activity to restore the BBB in the context of hypoxia‐induced angiogenic edema and thus reduce CSD.^66^, ^67^, ^68^ Moreover, we observed a decrease in some adhesion proteins in the occipital region of PFO‐positive mice. Reductions in the expression of adhesion molecules in neurons, such as PPM1H, NTSR2, CLDN10, and CDH13, are related to synaptic N‐methyl‐D‐aspartate (NMDA) receptor‐induced long‐term potentiation^69^ and affect serotonin (5‐HT) formation, which is potentially relevant to migraine.^70^, ^71^ In addition, previous studies have shown that changes in the levels of CNTN1, CDH1, and other cell adhesion proteins in peripheral blood are associated with deficit schizophrenia, which may also be related to hypoxia.^72^, ^73^ We also found an increase in CAMs in blood after PFO closure; however, the clinical implications of this change remain to be explored in the future study.

In this study, we also found that proteins related to mitochondrial organization changed after PFO closure, meanwhile the brain of PFO‐positive mice also showed enrichment of electron transfer and GTPase activity. These energy metabolic processes are probably associated with pathogenesis of migraine and epilepsy.^74^, ^75^ In subgroup analysis of epilepsy and migraine, we found proteins related to pyruvate metabolism increased after PFO closure in the peripheral blood of PWEs. Previous studies have found that pyruvate metabolism disorder related to epilepsy not only provides energy for the brain, but also plays a brain protective role.^76^, ^77^, ^78^ In addition, we also found angiogenesis‐related proteins increased, while retinol metabolism‐related proteins decreased in peripheral blood of PWMs. Migraine is closely associated with cerebrovascular damage and dysfunction and the elevation of angiogenesis‐related proteins may help to repair cerebrovascular.^79^, ^80^ The level of proteins associated with retinol metabolism was related to BBB dysfunction and vasculitis.^81^, ^82^ How these proteins regulate the progression of migraine and epilepsy should be further investigated. Moreover, higher‐level evidence, such as randomized controlled trials on PFO closure for the treatment of NCDEMs, is required to validate our findings in the future.

There are also some limitations in this study. Although we have demonstrated that PFO closure is beneficial for migraine and epilepsy improvements, these results need to be validated in a multicenter cohort with larger sample sizes. Besides, we did not repeat RLS screen after PFO closure, however, the association between the absence of symptom relief and residual shunt also deserves attention in the future. Additionally, further experimental authentication is required to verify this study. The use of free hemoglobin and CAMs to predict the prognosis of PWMs and PWEs is also a direction for future efforts.

## 4 CONCLUSION

In summary, we presented that PFO closure could improve the epilepsy and migraine by relieving hypoxia state. Multisite and multitimepoint proteomics indicated that hypoxia‐associated proteome restored after PFO closure and altered the expression of adhesion molecules in the occipital region, which may be responsible for PFO‐induced neurologic ictal disorders.

## 5 METHODS AND MATERIALS

### 5.1 Participant recruitment, evaluation, and follow‐up

PWEs or PWMs aged 9−55 years were recruited from the Neurology Department of the West China Hospital of Sichuan University between July 2018 and May 2022. Our diagnostic criteria for epilepsy and migraine were based on the International League Against Epilepsy (ILAE) 2014 criteria^83^ and the guidelines of the International Classification of Headache Disorders (ICHD‐3 beta),^84^ and all patients were diagnosed by two specialists independently. Patients were excluded if their epilepsy was due to a tumor, trauma or neurotoxicity; if they had any other structural heart diseases or cardiac valvular diseases; or if they exhibited any other distinct clinical diseases or syndromes. After informed consent was obtained, demographic and clinical information was recorded by specialists during interview, including the number of seizure or migraine attacks per month, aura, duration of seizure or headache, usage of ASM or headache remedies, and indicators of severity such as the Visual Analog Scale (VAS) for headache pain and the Migraine Headache Index Score (MHIS)^85^ were also calculated. Intractable epilepsy was identified as failure of adequate trials of two tolerated and appropriately chosen and used ASM schedules to achieve sustained seizure freedom according to 2010 ILAE definition^86^ and intractable migraine was identified as migraine headache on at least 8 days per month according to ICHD‐3 beta.^84^

Then, the recruited patients underwent contrast‐transthoracic echocardiography (cTTE) at the first visit after enrollment with a Philips IE 33 ultrasound system with 1−5 or 3−8 MHz multiplane transducers. Microbubble contrast medium mixed with an agitated solution of 8 mL of saline, 1 mL of blood, and 1 mL of air was injected as a bolus into the cubital vein for increased sensitivity.^87^ The PFO diagnosis were performed by two experienced sonographers based on the American Guidelines for the Echocardiographic Assessment of Atrial Septal Defect and Patent Foramen Ovale.^87^ RLS were assessed at rest, during a Valsalva maneuver and coughing during cTTE. The degree of RLS was quantified based on the number of detected microvesicles per frame in the left atrium: grade 0 = no occurrence of microbubbles; grade I = 1−10 microbubbles; grade II = 11−30 microbubbles; grade III = over 30 microbubbles, or left atrium nearly filled with microbubbles, or left atrial opacity.^87^ If the PWEs or PWMs diagnosed with PFO had an RLS more severe than grade II, they were referred to the Cardiology Department. Two experienced interventional cardiologists evaluated the transesophageal echocardiography results of patients who intended to undergo PFO closure and assessed the clinical indications for transcatheter PFO closure according to consensus among Chinese experts.^88^

After these evaluations, the cardiologists performed the interventional operation for PFO closure. For this operation, the patients were placed in the supine position on the operating table with local anesthesia, and the femoral vein was punctured. A sheath with a guide wire was inserted into the right atrium, and 3 mL of blood was collected from the right atrium. After entering the left atrium through the unclosed foramen ovale, 3 mL of blood was collected from the left atrium. The occluder was released after echocardiography confirmed the proper positioning and shape of the occluder and the absence of an apparent residual shunt. Three microliters of blood were taken from the right atrium before the arterial sheath was removed. Chest radiography and TTE were the main modalities used after the procedure to confirm that the devices were in a good position.

The monthly follow‐up evaluations consisted of outpatient visits with neurologists and telephone assessments administered by neurologists for more than one year. Follow‐up data included the number of seizure or migraine attacks per month, clinical manifestations, duration of seizure or headache, VAS score, MHIS, usage of ASM or headache remedies, and adverse effects, including chest pain, atrial fibrillation, paroxysmal supraventricular tachycardia, thrombosis, aortic dissection, occluder out of position, and any other related problem. Before and after operation in one year, 3 mL of peripheral blood from patients who underwent successful PFO closure was collected into standard ethylenediaminetetraacetic acid‐containing collection tube (Yuli). Fasting venous blood samples were collected during interictal period and processed within 30 min of collection. Plasma was collected after centrifugation (1800 g for 15 min at 4°C) of the blood samples. After venous blood collected, patients were asked to finish invasive arterial blood collection from the radial arteries and arterial blood was immediately analyzed using an on‐site blood gas analyzer (Cobas b123). All patients had no seizures during the day before and day of the blood collection.

This study complied with the declaration of Helsinki and was approved by the Ethics Committee of Sichuan University (ChiCTR‐OOC‐17011935 and ChiCTR2000031591).

### 5.2 Animals and sample collection

Breeding pairs of 129T2/SvEms mice with a PFO incidence of approximately 75% were purchased from the Jackson Laboratory.^89^ Mice were housed and bred in the animal SPF facility of Sichuan University. All animals were group‐housed (4 per cage) with food and water under a 12 h dark–light cycle (8:00 p.m. to 8:00 a.m.) in a temperature‐controlled room. Mice were anesthetized with 2−3% isoflurane inhalation. Retro‐orbital blood was collected from 6‐ to 8‐week‐old offspring after removing eyeballs, and the plasma was separated by low‐speed centrifugation (3000×*g* × 15 min). After rapid decapitation of the mice, the brains were rapidly removed, and the cerebral tissues were cut into three equally spaced (approximately 3 mm) coronal blocks stored at −80°C. Whole hearts were dissected, fixed in formalin (Solarbio) for 48 h, and embedded in wax. Next, 3.5 μm continuous coronal sections of the heart were stained with hematoxylin and eosin (H&E) to assess the presence of PFO according to a previously described procedure.^90^ The animal experiments were approved by the Institutional Animal Care and Use Committee of Sichuan University (20211503A) and conformed to the Guide for the Care and Use of Laboratory Animals.

### 5.3 LC‒MS/MS analysis of plasma samples from patients and mice

For proteome analysis, five microliters of plasma were thawed at 4°C, and 50 mM ammonium bicarbonate solution was added to obtain a total volume of 100 μL. The samples were then heated at 95°C for 3 min and cooled to room temperature before trypsin was added for enzymolysis at 37°C for 16 h. After the peptides were desalted and dried (SpeedVac, Eppendorf), they were reconstituted with 100 μL of 0.1% formic acid solution. Upon injection, the samples were separated in an Easy‐nLC1200 LC system (Thermo Fisher Scientific) coupled to a nanoelectrospray ion source (Thermo Fisher Scientific) and an Orbitrap Q Exactive HF‐X mass spectrometer (Thermo Fisher Scientific). Buffer A was a 0.1% formic acid aqueous solution, and buffer B contained 0.1% formic acid and 80% acetonitrile in an aqueous solution. The chromatography column was equilibrated with 100% buffer A, and the samples were loaded onto the analytical column by the autosampler (8 cm/ID 75 μm/1.9 μm/C18; Dr. Maisch GmbH) and separated at a flow rate of 600 nL/min. After chromatographic separation, the samples were analyzed by MS using data‐independent acquisition (DIA), which consisted of MS1 scans from 300 to 1400 m/z at 60k resolution (AGC target, 3e6; maximum IT, 20 ms). Then, 30 DIA segments were acquired at 15,000 resolutions. The “inject ions for all available parallelizable time” setting was enabled. HCD fragmentation was set to a normalized collision energy of 30%, and the resolution of MS/MS analysis was 15,000 at 200 m/z.

### 5.4 LC‒MS/MS analysis of brain tissue samples from mice

Approximately 10 mg of brain tissue was taken from frontal, temporal, occipital regions of each mouse, weighed on ice, transferred into a 1.5 mL EP tube, and washed several times with PBS. TCEP solution containing protease and phosphatase inhibitors were added to each sample. After grinding the brain tissues, they were lysed by ultrasonication, and proteins were extracted by centrifugation (4°C, 8000×*g* × 2 min). The protein concentrations in the supernatants were measured by the Bradford assay. An appropriate amount of trypsin (1:25, w/w) was then added to each sample, and the samples were digested overnight. The digested peptides were dried, redissolved with 0.1% formic acid aqueous solution, and desalted with C18 desalting tips.

The samples were reconstituted with 100 μL of 0.1% formic acid solution and analyzed using the Easy‐nLC1200 HPLC system (Thermo Fisher Scientific) coupled to a nanoelectrospray ion source (Thermo Fisher Scientific) and an Orbitrap Q Exactive HF‐X mass spectrometer (Thermo Fisher Scientific). A precolumn (2 cm/ID 100 μm/3 μm/C18; Dr. Maisch GmbH) and an analytical column (30 cm/ID 150 μm/1.9 μm/C18; Dr. Maisch GmbH) were used. The samples were separated at a flow rate of 600 nL/min. The elution gradient was as follows: after an initial concentration of 6% buffer B, the concentration was increased to 12% over 8 min; then, the concentration was further increased from 13 to 30% over the next 92 min, increased to 45% over 10 min, quickly increased to 100% over 1 min, and finally held at 100% for 9 min. The peptide samples were analyzed with a Q Exactive HF‐X mass spectrometer. The full scans were performed from 300 to 1400 m/z at 120,000 resolutions, and the AGC target was set at 3e6. Sixty precursor ions were fragmented in the MS2 analysis. Higher‐energy collisional dissociation mode with a normalized collision energy of 27% was used. The isolation window was 1.6 m/z, and the MS/MS analysis resolution was 7500 at 200 m/z.

### 5.5 MS data search

MS raw files performed for both plasma and brain samples were processed with the Firmiana proteomics workstation.^91^ Proteins from human were compared with the UniProt human protein database (version 2019.12.17, 12732 entries) using the integrated Mascot 2.7 search engine and proteins from mice were identified against the RefSeq mouse protein database (version 2013.04.07, 7750 entries) using the Mascot 2.7 search engine.^92^, ^93^ Mass tolerances of 20 ppm for precursor and 0.5 Da for production were allowed. The maximum number of missed cleavages was set to 2. The search engine set cysteine carbamicdomethylation as fixed modification, and N‐acetylation and oxidation of methionine as variable modifications. For the QC of protein identification, false discovery rate was lower than 1%. The one‐step proteomic cloud platform “Firmiana” was further employed for protein quantification. All the protein quantifications were calculated using a label‐free, intensity‐based absolute quantification (iBAQ) approach.^94^ The fraction of total, a relative quantification value defined as a protein's iBAQ divided by the total iBAQ of all identified proteins in one experiment, was used to represent the normalized abundance of a particular protein across samples.

### 5.6 RNA extraction from mouse brain tissue and library construction and sequencing

Total RNA was extracted from mouse brain tissues using the RNeasy Plus Universal Mini Kit (Qiagen) following the manufacturer's instructions. RNA quantity and quality were assessed using a Qubit Flex fluorometer (Invitrogen) and an Agilent 2100 Bioanalyzer (Agilent Technologies) prior to library construction and sequencing. The effective library concentration was accurately quantified by qRT‐PCR to ensure quality (an effective library concentration was higher than 2 nM). Then, the different libraries were pooled according to the effective concentration and requirements of the target data volume. RNA sequencing was performed on an Illumina NovaSeq 6000, and 150 bp paired‐end reads were generated. The sequencer captured the fluorescent signals from dNTPs and converted the optical signals into sequence information from the fragments.

### 5.7 Bioinformatics and statistical analysis

Proteomics data were obtained for five groups of human plasma samples: preoperative left atrial plasma, preoperative right atrial plasma, preoperative peripheral plasma, postoperative right atrial plasma, and postoperative peripheral plasma. The mouse proteomics data were grouped according to sample type (plasma from peripheral blood or from the occipital region, temporal region, or frontal region), and the samples in each group were divided into the PFO‐positive and PFO‐negative subgroups according to the cardiac H&E staining results. All proteomics data were filtered for missing values in each group, and proteins with less than 50% missing values within groups were retained. As a result, the total numbers of proteins obtained were as follows: 1834 in human plasma, 1249 in mouse plasma, 4004 proteins in the occipital region, 4048 in the temporal region, and 4148 in the frontal region. Missing values in all the proteomics datasets were estimated using the KNN method in the R package DMwR2, and the normality of these datasets was tested with the R package nortest.

In the human plasma proteomics dataset, data for only 7.4% of proteins conformed to a normal distribution, and this percent increased to 14.9% after log_2_ transformation. While data for 58.3% of proteins in mouse plasma conformed to a normal distribution, this percentage increased to 72.2% after log_2_ transformation. Similarly, data for 78.4% of proteins in the occipital dataset conformed to a normal distribution, and this proportion increased to 81.9% after log_2_ transformation; in the temporal region dataset, data for 70.2% of the proteins conformed to a normal distribution, and this percentage increased to 82.6% after log_2_ transformation; and in the frontal region dataset, data for 76.5% of the proteins conformed to a normal distribution, and this percentage increased to 82.6% after log_2_ transformation. Therefore, nonparametric tests were utilized to analyze the human proteomics data; specifically, the Mann‒Whitney *U* test was used to identify differentially expressed proteins between two groups, and the Kruskal‒Wallis test was used to identify differentially expressed proteins among more than two groups. Log_2_ transformed data for mouse samples were analyzed by Student's *t*‐test. Significant differential protein expression between the two groups was indicated by *p* < 0.05 and fold change > 1.2. Similarly, *p* < 0.05 was considered to indicate statistical significance in comparisons of multiple groups.

Protein cluster analysis was performed using proteomics data for the preoperative left atrial plasma, preoperative right atrial plasma and postoperative right atrial plasma groups. The Kruskal‒Wallis test was used to identify differentially expressed proteins among the above three groups. The median level of each differentially expressed protein in the three groups was calculated. Afterward, the differentially expressed proteins were clustered according to the change in the median using the R package pheatmap (the clustering parameter was set to “ward. D”), and kmeans was used to assess the number of classifications. Then, these proteins were classified by the cutree function. The functional enrichment of differentially expressed proteins was carried out by String (11.5) and Metascape.^95^ The PPI analysis of differentially expressed protein was performed based on the STRING online database (https://string‐db.org) and visualized in Cytoscape software.

For the transcriptome analysis of samples from the mouse occipital, temporal, and frontal regions, flanking and low‐quality sequences were removed by Trimmomatic. RNA‐seq reads were mapped to the mouse reference genome (mm10/GRCm38) using STAR and quantified by featureCounts. Deseq2 was used for differential expression analysis, and *p* values were calculated using a negative binomial distribution as a model for expected gene expression. A *p* value < 0.05 and |log_2_ (fold change)| ≥ 0.585 were used as thresholds for significant differences in gene expression.

## AUTHOR CONTRIBUTIONS

*Conception*: Bosi Dong, Wanling Li, Yucheng Chen, Lunzhi Dai, and Lei Chen. *Acquisition*: Bosi Dong, Yajiao Li, Qi Lai, Wanling Li, Yucheng Chen, and Lei Chen. *Analysis*: Ying Lu, Siyu He, Bosi Dong, Baichuan Li, Shuming Ji, Yunwu Zhang, and Lunzhi Dai. *Drafting*: Bosi Dong, Ying Lu, Lunzhi Dai, and Lei Chen. *Revising*: Bosi Dong, Ying Lu, Lunzhi Dai, and Lei Chen. *Final approval*: Bosi Dong, Ying Lu, Siyu He, Baichuan Li, Yajiao Li, Qi Lai, Wanling Li, Shuming Ji, Yucheng Chen, Lunzhi Dai, and Lei Chen. All authors have read and approved the final manuscript.

## CONFLICT OF INTEREST STATEMENT

The authors declare no conflicts of interest.

## ETHICS STATEMENT

This study involving human participants complied with the declaration of Helsinki and was approved by the Ethics Committee of Sichuan University (No. 201779 and 202058). The clinical study was registered at the Chinese Clinical Trial Register (ChiCTR‐OOC‐17011935 and ChiCTR2000031591). *The research on the relationship of patent foramen ovale and epilepsy*, ChiCTR‐OOC‐17011935, registered July 10, 2017, http://www.chictr.org.cn/showproj.aspx?proj = 20071; *Patent foramen ovale closure for epilepsy: a study based on genomics, proteomics and fMRI*, ChiCTR2000031396, registered March 30, 2020, http://www.chictr.org.cn/showproj.aspx?proj = 50816. All patients provided written informed consent. This study involving mice was approved by the Institutional Animal Care and Use Committee of Sichuan University (No. 20211503A) and conformed to the Guide for the Care and Use of Laboratory Animals.

## Supporting information

Supporting Information

## Data Availability

The datasets used and/or analyzed during the current study are available from the corresponding author on reasonable request.
