# Supplementary material for: Multisite and multitimepoint proteomics reveal that patent foramen ovale closure improves migraine and epilepsy by reducing right‐to‐left shunt‐induced hypoxia
Source: MedComm (2020). 2023 Aug 12;4(4):e334. doi: 10.1002/mco2.334 (PMC10422075; doi:10.1002/mco2.334)
Supplement: Supplementary file 1 — Supporting Information [file MCO2-4-e334-s001.docx]

**Multi-site and multi-timepoint proteomics reveal that patent foramen ovale closure improves migraine and epilepsy by reducing right-to-left shunt-induced hypoxia**

Bosi Dong^1#^, Ying Lu^2#^, Siyu He^2^, Baichuan Li^1^, Yajiao Li^3^, Qi Lai^1^, Wanling Li^1^, Shuming Ji^4^, Yucheng Chen^3^, Lunzhi Dai^2*^, Lei Chen^1*^

^1^Department of Neurology, West China Hospital, Sichuan University, Chengdu, 610044, Sichuan, China

^2^National Clinical Research Center for Geriatrics and Department of General Practice, State Key Laboratory of Biotherapy, West China Hospital, Sichuan University, and Collaborative Innovation Center of Biotherapy, Chengdu, 610041, Sichuan, China.

^3^Department of Cardiology, West China Hospital, Sichuan University, Chengdu, 610044, Sichuan, China.

^4^Department of Clinical Research Management, West China Hospital, Sichuan University, Chengdu, 610044, Sichuan, China

**Supplementary tables**

**Table S1.** The significant GO terms enriched in cluster analysis based on STRING database.

| term ID | description | observed gene count | background gene count | strength | false discovery rate |
| --- | --- | --- | --- | --- | --- |
| GO:0003824 | Catalytic activity | 17 | 5486 | 0.42 | 0.0216 |
| GO:0031721 | Hemoglobin alpha binding | 3 | 5 | 2.96 | 4.14E-05 |
| GO:0005344 | Oxygen carrier activity | 3 | 14 | 2.51 | 0.00017 |
| GO:0019825 | Oxygen binding | 3 | 36 | 2.1 | 0.0017 |
| GO:0043177 | Organic acid binding | 4 | 230 | 1.42 | 0.0085 |
| GO:0020037 | Heme binding | 3 | 134 | 1.53 | 0.0491 |

**Table S2.** GO analyses of differentially expressed protein in blood sample of PFO closure by Metascape.

| term ID | description | LogP | Log(q-value) | hitlist |
| --- | --- | --- | --- | --- |
| GO:0009636 | response to toxic substance | -3.568281 | 0.000 | 3/229 |
| GO:0043270 | positive regulation of ion transport | -3.345024 | 0.000 | 3/273 |
| GO:0016071 | mRNA metabolic process | -5.113209 | -0.767 | 7/606 |
| GO:0031347 | regulation of defense response | -3.90117 | -0.140 | 6/648 |
| GO:0098609 | cell-cell adhesion | -3.338464 | 0.000 | 5/533 |
| GO:0007160 | cell-matrix adhesion | -3.245462 | 0.000 | 3/132 |
| GO:0044272 | sulfur compound biosynthetic process | -3.171151 | 0.000 | 3/140 |
| GO:0051098 | regulation of binding | -2.991161 | 0.000 | 4/366 |
| GO:0080135 | regulation of cellular response to stress | -2.830712 | 0.000 | 5/692 |
| GO:0034097 | response to cytokine | -2.558722 | 0.000 | 5/799 |
| GO:0043588 | skin development | -4.138206 | 0 | 4/235 |
| GO:0071345 | cellular response to cytokine stimulus | -3.298822 | 0 | 5/703 |
| GO:0009611 | response to wounding | -3.139606 | 0 | 4/430 |
| GO:0097435 | supramolecular fiber organization | -2.828167 | 0 | 4/522 |
| GO:0031330 | negative regulation of cellular catabolic process | -2.695535 | 0 | 3/263 |
| GO:0007167 | enzyme linked receptor protein signaling pathway | -2.605393 | 0 | 4/601 |
| GO:0051129 | negative regulation of cellular component organization | -2.40398 | 0 | 4/684 |
| GO:0043086 | negative regulation of catalytic activity | -2.191304 | 0 | 4/786 |
| GO:0050804 | modulation of chemical synaptic transmission | -2.105132 | 0 | 3/428 |
| GO:0032516 | positive regulation of phosphoprotein phosphatase activity | -8.732895 | -4.671 | 4/22 |
| GO:0042060 | wound healing | -5.390406 | -3.049 | 5/326 |
| GO:0060348 | bone development | -4.842396 | -2.646 | 4/198 |
| GO:0009152 | purine ribonucleotide biosynthetic process | -3.756646 | -1.778 | 3/144 |
| GO:0098609 | cell-cell adhesion | -3.187192 | -1.298 | 4/533 |
| GO:0001775 | cell activation | -2.902063 | -1.041 | 4/636 |
| GO:0007005 | mitochondrion organization | -2.421206 | -0.598 | 3/418 |
| GO:0098662 | inorganic cation transmembrane transport | -2.046856 | -0.239 | 3/571 |

**Table S3.** Clinical characters of patients with pre- and post-operative blood gas analysis.

|  | Patients underwent blood gas analysis |
| --- | --- |
| Gender （male,%） | 0 (0.0) |
| Onset age (y, mean±SD) | 30.8 ± 11.90 |
| Age at PFO closure (y, mean±SD) | 41.20 ± 9.20 |
| RLS grade (III, %) | 5 (100.0) |
| With Aura (n, %) | 0 (0.0) |
| Intractable migraine or epilepsy (n, %) | 1 (20.0) |
| More than 50% reduction in frequency of attacks (n, %) | 2 (40.0) |

**Table S4.** GO analyses of differentially expressed protein in molecular function of PFO occipital region by String.

| term description | observed gene count | background gene count | strength | false discovery rate |
| --- | --- | --- | --- | --- |
| Oxidoreductase activity | 79 | 726 | 0.43 | 5.64E-12 |
| Cadherin binding | 46 | 334 | 0.54 | 9.77E-10 |
| GTPase activity | 42 | 318 | 0.52 | 2.42E-08 |
| Nucleoside binding | 46 | 384 | 0.48 | 5.69E-08 |
| GTP binding | 45 | 370 | 0.48 | 5.69E-08 |
| Cell adhesion molecule binding | 55 | 538 | 0.41 | 1.88E-07 |
| NADH dehydrogenase (ubiquinone) activity | 15 | 46 | 0.91 | 7.19E-07 |
| Electron transfer activity | 18 | 103 | 0.64 | 7.05E-05 |
| NAD binding | 13 | 56 | 0.76 | 0.00015 |
| Translation factor activity, RNA binding | 15 | 82 | 0.66 | 0.00032 |
| Oxidoreductase activity, acting on NAD(P)H | 17 | 107 | 0.6 | 0.00039 |
| Translation regulator activity, nucleic acid binding | 16 | 101 | 0.6 | 0.00073 |

**Supplementary Figures**

**
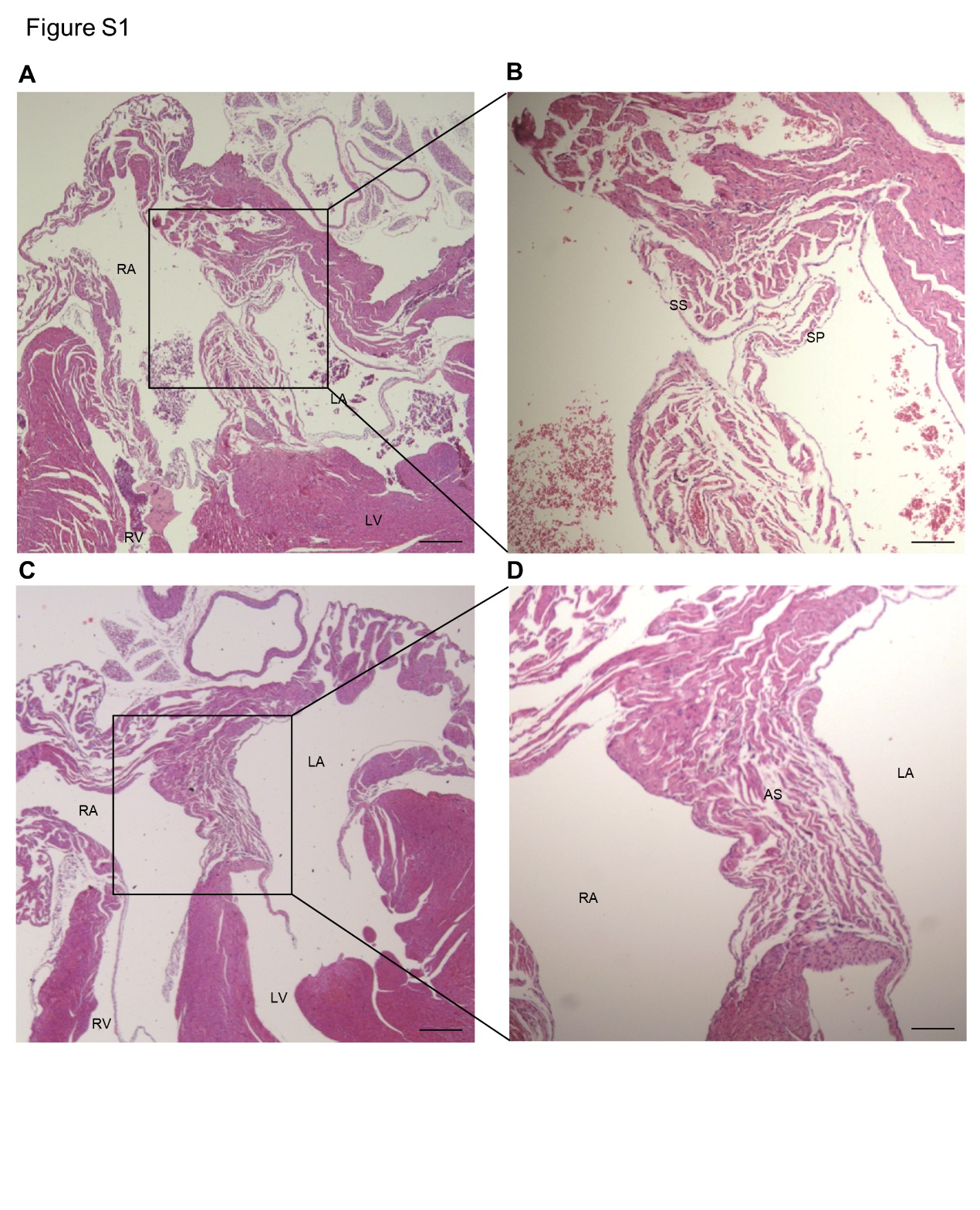
**

**Figure S1. H&E stains of heart structure in PFO mouse models.** A-B. Representative histological heart section demonstrating open foramen. C-D. Representative histological heart section demonstrating closed foramen ovale. Bar = 400 µm (A, C). Bars in other panels = 100 µm. PFO, patent foramen ovale; AS, atrial septum; RA, right artium; LA, left atrium; RV, right ventricle; LV, left ventricle; SP, septum primum; SS, septum secundum.


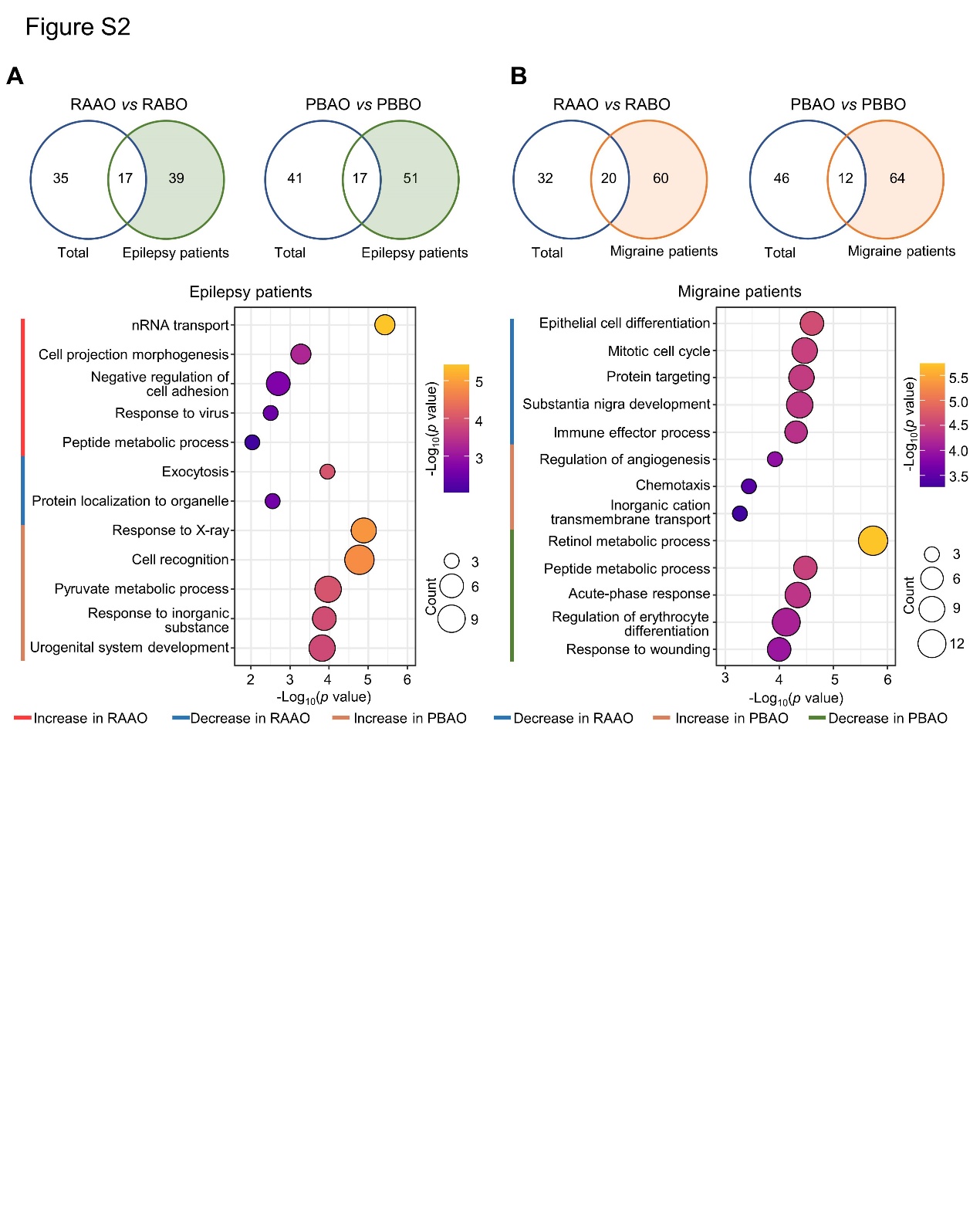


**Figure S2. Subgroup analyses of epilepsy and migraine.** A. Venn diagraphs of differentially expressed proteins from right atrium and peripheral veins of all NCDEMs and PWEs and enrichment analysis of differentially expressed proteins after PFO closure from right atrium and peripheral veins of PWEs. B. Venn diagraphs of differentially expressed proteins from right atrium and peripheral veins of all NCDEMs and PWMs and enrichment analysis of differentially expressed proteins after PFO closure from right atrium and peripheral veins of PWMs. NCDEM, neurological chronic diseases with episodic manifestation; PWE, patient with epilepsy; PWM, patient with migraine; RABO, blood of the right atrium before the operation; RAAO, blood of the right atrium after the operation; PBBO, peripheral blood before operation; PBAO, peripheral blood after operation.


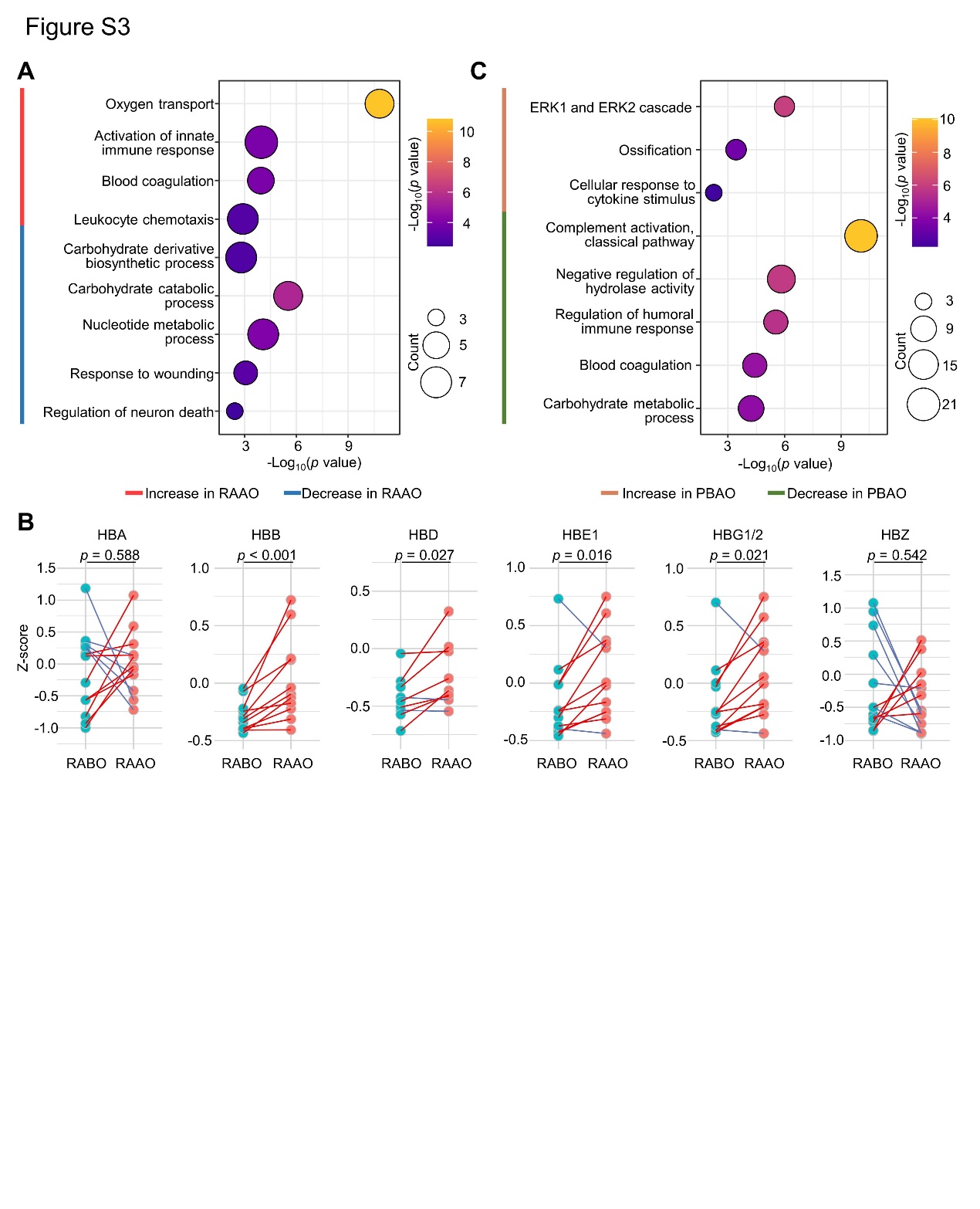


**Figure S3. Subgroup analyses of improvers and non-improvers.** A. Enrichment analysis of differentially expressed proteins of improvers in right atrium. B. The plot presents the levels of free hemoglobin variants in the right atrium blood and each line connects the same improvers before and after PFO closure. C. Enrichment analysis of differentially expressed proteins of improvers in peripheral blood. RABO, blood of the right atrium before the operation; RAAO, blood of the right atrium after the operation; PBBO, peripheral blood before operation; PBAO, peripheral blood after operation.
